# BOK beyond apoptosis: pyrimidine metabolism and ATR dependence in p53-deficient lung cancer

**DOI:** 10.1038/s41418-026-01687-9

**Published:** 2026-02-11

**Authors:** Nicoletta Franco, Licio Collavin

**Affiliations:** Department of Life Sciences, University of Trieste, Trieste, Italy

**Keywords:** Cancer metabolism, Lung cancer

Members of the Bcl-2 (B Cell lymphoma 2) protein family are key regulators of the intrinsic apoptosis pathway triggered by the assembly of permeability pores on the mitochondrial outer membrane (MOM). The family lists more than 15 proteins, functionally divided into pro-apoptotic or anti-apoptotic based on their activity and structural features [1].

BOK (Bcl-2-related ovarian killer) is listed as a pro-apoptotic member of the family, together with Bak and Bax, because it can induce apoptosis when overexpressed in various cell types and can permeabilize artificial MOM models in vitro. However, many evidences indicate that BOK has relevant apoptosis-independent functions, and some authors even challenge the assumption that Bok is a “killer” [2, 3].

Differently than other Bcl-2 family proteins, BOK is localized on the endoplasmic reticulum (ER), where it stably interacts with inositol 1,4,5 trisphosphate receptor (IP3R) subunits. The functional relevance of this interaction is still uncertain, but BOK has been implicated in the cellular response to ER stress and in controlling mitochondrial dynamics and the stability of mitochondria-associated ER membranes [2, 3].

In addition, Bok interacts with uridine monophosphate synthetase (UMPS) [4]. UMPS is a cytoplasmic bifunctional enzyme that catalyzes the final steps in the conversion of orotate to uridine monophosphate (UMP). UMPS catalyzes a rate-limiting step in the de novo synthesis of pyrimidine nucleotides, and although UMP can be produced from uridine or cytidine via a salvage pathway, cells with a high proliferative rate, such as cancer cells, are largely dependent on the de novo synthesis pathway [5].

BOK interacts with UMPS via its BH3 domain and stimulates the activity of the enzyme up to 3-fold in vitro [4]. As a consequence, BOK inactivation/deletion is expected to reduce the rate of de novo pyrimidine synthesis. In cancer cells, this has two effects: on one hand, it slows cell proliferation because nucleotide imbalance triggers replication stress; on the other hand, it increases chemoresistance to 5-fluorouracil (5-FU) because UMPS is the enzyme that converts 5-FU into toxic metabolites. Accordingly, it was reported that BOK is frequently downregulated or deleted in NSCLC patients, with BOK protein levels positively correlated with survival [6]. Similarly, colorectal cancer cells downregulate BOK expression and gain resistance to 5-FU, possibly acquiring a selective advantage during tumor progression [4, 7].

A study by JeanRichards et al. in this issue provides additional evidence for the relevance of BOK/UMPS interaction in the context of lung cancer [8]. They used a human NSCLC cell line in which BOK and/or p53 were deleted by CRISPR/CAS9. They also deleted BOK in two NSCLC cell lines derived from mouse tumors driven by conditional activation of Ras and Myc oncogenes; notably, one of the two lines had spontaneously mutated p53, thus recapitulating a frequent event in human cancers. These models allowed authors to test the impact of BOK depletion in the presence or absence of functional p53.

They confirmed that BOK loss reduced proliferation of cells with wt p53, while no proliferation defects were observed in p53 KO or p53 mutated cells. However, BOK deletion in these cells had a phenotype: it increased baseline DNA damage.

The authors provide convincing evidence that DNA damage in BOK-deleted cells is related to UMPS functions. First of all, treatment with a cell-permeable TAT peptide carrying the BH3 domain of BOK, the region that binds UMPS and presumably stimulates its enzymatic activity, was sufficient to rescue the increased DNA damage in BOK–/– cells. Second, the increased DNA damage in BOK –/– cells was rescued by medium supplementation with UMP and CMP, bypassing the requirement for de novo pyrimidine synthesis [8].

Imbalance of nucleotides induces DNA damage by replication stress in oncogene-expressing cells [9, 10]; it is plausible that BOK deletion diminishes UMPS activity and de novo pyrimidine synthesis to an extent sufficient to cause replication stress and DNA damage. In the presence of a functional p53, this condition triggers cell cycle arrest. In the absence of p53, DNA lesions induced by replication stress accumulate, and cells become more dependent on DNA damage repair and the ATR/CHK1 pathway.

**Fig. 1:**
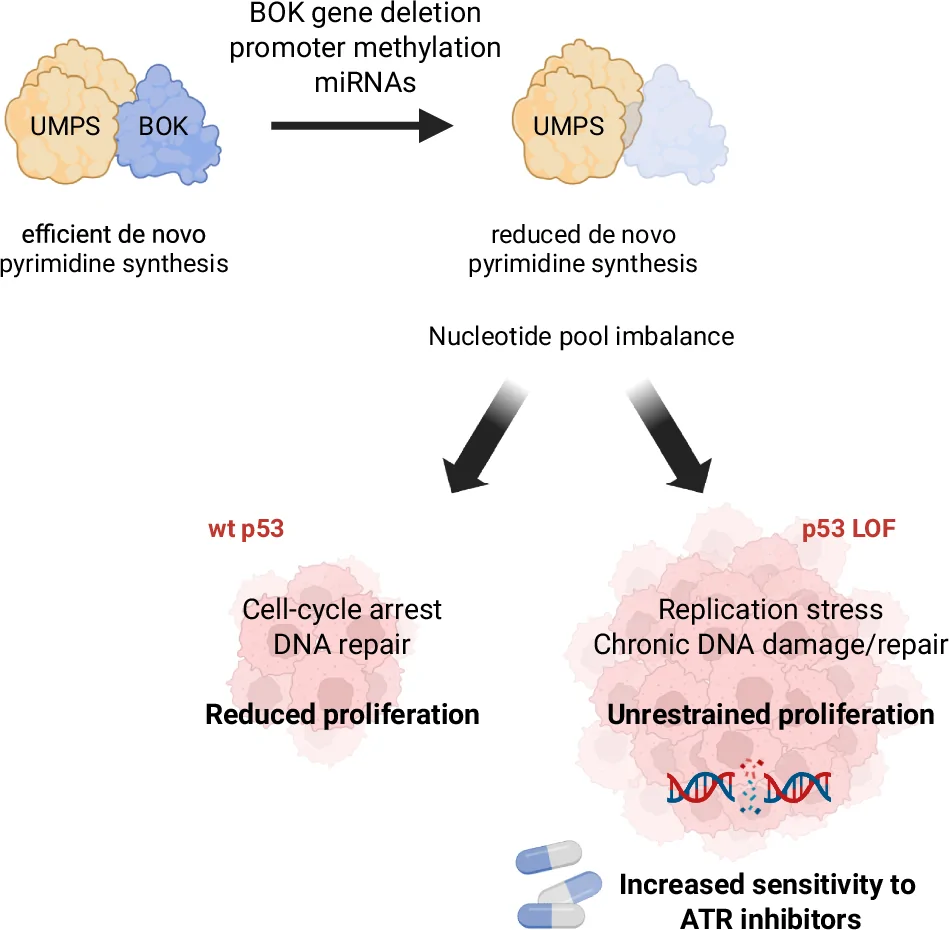
BOK interaction with UMPS stimulates its enzymatic activity and de novo synthesis of UMP. In tumors, BOK can be inactivated by chromosomal deletions, promoter methylation, or microRNA-mediated repression. In the absence of BOK, UMPS activity is reduced, causing an imbalance in the pyrimidine/purine ratio and a p53-dependent proliferation arrest. In cells with p53 loss of function (p53 LOF), nucleotide imbalance causes chronic replication stress and DNA damage, and they become highly dependent on ATR function (created with BioRender.com).

JeanRichard and colleagues treated BOK/p53 double knockout cells with the drug ceralasertib (AZD6738), a specific ATR inhibitor [11]. In their models, ceralasertib further increased DNA damage and caused cell death more efficiently in BOK/p53 double knockout cells as compared to BOK KO cells with wtp53, revealing a specific sensitivity of p53-deficient cells to ATR inhibition (Fig. 1). This finding has attractive implications, since ATR inhibitors are currently undergoing clinical trials in advanced solid tumors, including NSCLC [11]; retrospective analysis of BOK and p53 status could provide valuable information to interpret the results of these studies, and perhaps guide therapeutic choices in the future.

The present study has some limits: it is based only on cultured cells and relies on BOK knockout, while in tumors BOK expression is often reduced but not abolished. Nonetheless, it convincingly links pyrimidine imbalance to a specific ATR dependence in the absence of functional p53. This may suggest a more general therapeutic line of intervention based on targeting UMPS to enhance sensitivity to ATR inhibitors in tumors with mutant p53. The UMPS inhibitor Pyrazofurin was tested in clinical studies in the late 1970s; despite good efficacy in preclinical models, clinical trials gave unsatisfactory results, and interest in this drug has rapidly faded [12, 13]. It might be worth reconsidering this therapeutic approach, taking into account the p53 status and the combination with ATR blockers. Eventually, the data by JeanRichard and colleagues may revitalize pharmacological research for novel and better UMPS inhibitors.
